# Electron beam effect on biomaterials I: focusing on bone graft materials

**DOI:** 10.1186/s40824-015-0031-5

**Published:** 2015-04-21

**Authors:** Soung Min Kim, Huan Fan, Yun Ju Cho, Mi Young Eo, Ji Hyun Park, Byung Nam Kim, Byung Cheol Lee, Suk Keun Lee

**Affiliations:** Department of Oral and Maxillofacial Surgery, Dental Research Institute, School of Dentistry, Seoul National University, 62-1 Changgyeonggungno, Jongno-gu, Seoul 110-768 South Korea; Quantum Optics Research Division, Korea Atomic Energy Research Institute, Daejeon, South Korea; Radiation Instrumentation Research Division, Korea Atomic Energy Research Institute, Daejeon, South Korea; Department of Oral Pathology, College of Dentistry, Gangneung-Wonju National University, 123 Chibyon-dong, Gangneung, 210-702 South Korea

**Keywords:** Allogenic bone, Xenogenic bone, Synthetic bone, Bony regeneration, Electron beam irradiation

## Abstract

**Background:**

To develop biocompatible bony regeneration materials, allogenic, xenogenic and synthetic bones have been irradiated by an electron beam to change the basic structures of their inorganic materials. The optimal electron beam energy and individual dose have not been established for maximizing the bony regeneration capacity in electron beam irradiated bone.

**Results:**

Commercial products consisting of four allogenic bones, six xenogenic bones, and six synthetic bones were used in this study. We used 1.0-MeV and 2.0 MeV linear accelerators (power: 100 KW, pressure; 115 kPa, temperature; -30 to 120°C, sensor sensitivity: 0.1-1.2 mV/kPa, generating power sensitivity: 44.75 mV/kPa, supply voltage: 50.25 V), and a microtrone with different individual irradiation doses such as 60 kGy and 120 kGy. Additional *in vitro* analyses were performed by elementary analysis using field emission scanning electron microscopy (FE-SEM), scanning electron microscopy (SEM), X-ray diffraction (XRD) and confocal laser scanning microscopy (CLSM). *In vivo* clinical, radiographic, and micro-computed tomography (Micro-CT) with bone marrow density (BMD) analysis was performed in 8- and 16-week-old Spraque-Dawley rats with calvarial defect grafts.

**Conclusions:**

Electron beam irradiation of bony substitutes has four main effects: the cross-linking of biphasic calcium phosphate bony apatite, chain-scissioning, the induction of rheological changes, and microbiological sterilization. These novel results and conclusions are the effects of electron beam irradiation.

## Background

Electron beam irradiation (EBI), or electron beam processing, is a process that uses electrons, usually of high energy, to treat objects for purposes such as sterilization and the cross-linking of polymers. Electron beams have been used in research, technology, and medical therapy to produce images on television screens, and are also used for electron microscopes for the ultramicroscopic analysis of materials [1-3].

**Figure 1:**
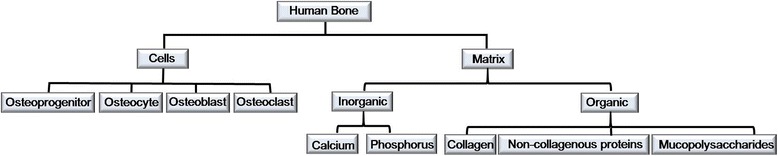
Schematic drawing of the basic compositions of human bone.

For the successful regeneration of grafted bone materials in the human body, many factors including bone composition and the bony matrix must be considered (Figure 1) [1,4]. The four basic desired properties of bone graft materials are osteogenesis, osteoinduction, osteoconduction, and osteointegration. Only autogenous bone has these four qualities, so many studies have been performed investigating the development of ideal bone graft materials from allogenic, xenogenic, and synthetic graft materials.

During our recent investigations of the effec of EBI on maxillofacial reconstructive polymer materials, we have tried to identify gross changes in the substitute bone materials. To advance the study of applying biomaterial in hard-tissue reconstruction, this article is intended to suggest new possibilities for electron beam treatment of bone graft materials.

## Methods

### Preparation of electron beam-irradiated bone

To prepare the electron beam-irradiated bone, we first obtained commercially available samples consisting of four allogenic bones, six xenogenic bones, and six synthetic bones. We used 1.0-MeV and 2.0-MeV linear accelerators (power 100 KW, pressure 115 kPa, temperature -30 to 120°C, sensor sensitivity: 0.1-1.2 m V/kPa, generating power sensitivity: 44.75 mV/kPa, supply voltage: 50.25 V), and a microtrone with different individual irradiation doses such as 60 kGy and 120 kGy.

The allogenic bone materials were Accell® (ISOTIS OrthogBiologics Inc., Irvine, USA), Allotis® (BioTis Bone Bank, Seoul, Korea), Oragraft® (LifeNet, Virginia Beach, VA, USA), and Orthoblast® (Integra Orthobiologics Inc., Irvine, USA). The xenogenic bone materials were BBP® (OscoTec Inc., Seongnam, Korea), Bio-cera® (OscoTec Inc., Seongnam, Korea), Bio-oss® (Geistlich Pharma AG, Wolhusen, Switzerland), Indu-cera® (OscoTec Inc., Seongnam, Korea), OCS-B® (Nibec Co., Seoul, Korea), and OCS-H® (Nibec Co., Seoul, Korea). The synthetic bone materials were BMP® (Cowellmedi Co., Seoul, Korea), BoneMedik® (Meta Biomed Co., Cheongju, Korea), Boneplus® (Megagen Co., Seoul, Korea), MBCP® (Biomatlante, vigneux de Bretagne, France), Osteon® (Genoss, Suwon, Korea), and Osteogen® (Impladent Ltd., Hollis, USA).

### *In vitro* analyses

**Figure 2:**
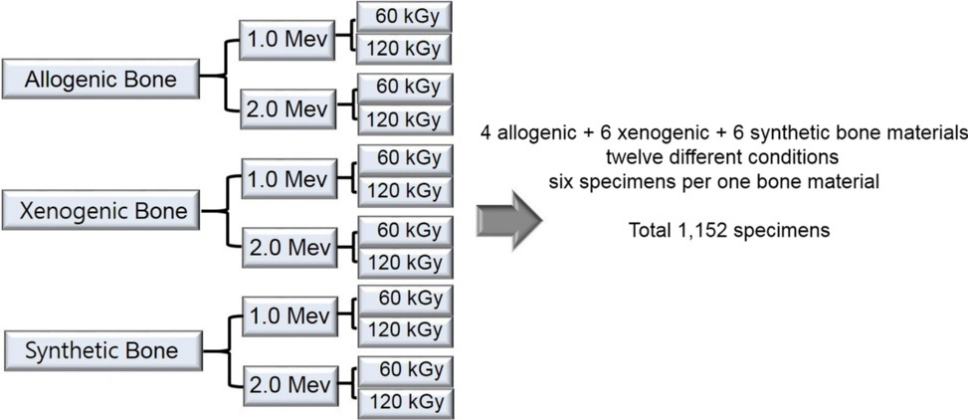
Basic study designs showing the electron beam irradiated bone materials, according to their origin, energy, and radiation dose.

After the irradiation of all specimens under strict dose calculations (Figure 2), all specimens were analyzed *in vitro* by elementary analysis with an EA 1110® elementary analyzer (CE instruments Co., Milan, Italy) using cold field emission. To observe the changed ultra-structures and to detect their elementary components, a Leo1455VP-SEM® (Carl Zeiss Inc., Aalen, Germany) scanning electron microscope (SEM), and S-4200® (Hitachi Co., Tokyo, Japan) field emission scanning electron microscope (FE-SEM) were also used. For the analysis of the molecular changes after EBI, 8D advance® (Bruker Co., Berlin, Germany) X-ray diffraction (XRD) analysis was performed. The three-dimensional changes on the surfaces of the specimen were evaluated with a LSM 5 Pascal® (Carl Zeiss Inc., Aalen, Germany) confocal laser scanning microscope (CLSM).

### Clinical and bone marrow density evaluation after the grafting of calvarial defects of Sprague-Dawley rat

**Figure 3:**
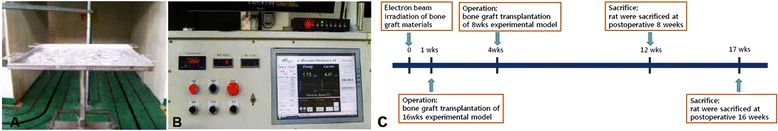
Basic units of the electron beam irradiation devices in EB Tech Co., Daejeon, South Korea. Specimens holding tables **(A)**, monitoring device, and the actual radiation dosages according to the set energy **(B)**, and schematic time-tables of the clinical, radiologic, and histologic study of a calvarial defect model grafted with electron beam-irradiated bone materials **(C)**.

We compared possible differences in each bone material before and after EBI, and each bone materials was implanted onto the perforated calvarial defects of Sprague-Dawley rats. Evaluation at 8 and 16 weeks was performed (Figure 3).

**Figure 4:**
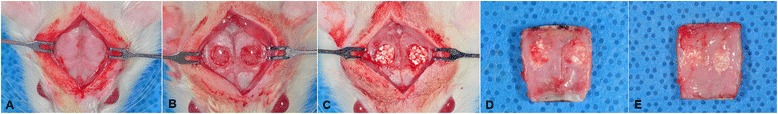
Implantation of each bone material and the evaluation process showing the exposed bilateral frontal bone of the Sprague-Dawley rat **(A)**, 5.0-mm-diameter perforations of the frontal bone were created with a round bur, avoiding damage to the internal brain tissues **(B)**, implantation of each perforated calvarial wound **(C)**, acquired frontal bone tissue including the perforated area and covering periosteal membrane after 8 weeks **(D)** and 16 weeks **(E)**.

In total, 48 Sprague-Dawley male rats (each weighing 200-230 g) were anesthetized with a mixture of Ketamine® (60 mg/kg, ketamine hydrochloride; Yuhan Co., Korea) and Rompun® (3 mg/kg, xylazine hydrochloride; Bayer Korea, Korea) in a 4:1 ratio. The rats’ bilateral frontal bones were exposed (Figure 4A) and perforated with a round bur (5.0 mm diameter; Shinhung Co., Korea) so as not to damage the internal brain tissue (Figure 4B). The result was a round bone perforation measuring 5 mm in diameter, and the perforated bone wound was filled with each bone graft materials (Figure 4C). After implantation, the wound was covered by a periosteal membrane suture using 5-0 Vicryl® (Johnson & Johnson Co., USA), followed by a tight suture of the incised skin with 4-0 Nylon® (Ailee Co., Korea). After surgery, animals were kept warm in their individual cages until they made a full recovery from the anesthesia and were then returned to the holding room with free access to water and food. Tarasyn® (2 mg/kg, ketorolac tromethamine; Yuhan Co., Korea) was given to each animal subcutaneously for 3 days to reduce any postoperative pain, and Icepacin® (1.5 mg/kg, icepacin sulfate; Yuhan Co., Korea) was used for 3 days to prevent postoperative infection.

The skin wounds healed uneventfully after two weeks. A group of rats was sacrificed by intramuscular injection of 50% Urethane® (U2500, 1.5 g/kg; Sigma-Aldrich Co., USA) at 8 (Figure 4D) and 16 weeks (Figure 4E) post-treatment. The frontal bone tissue, including the perforated area and covering periosteal membrane, was removed (Figures 4D,E). Each removed specimen was evaluated with micro-computed tomography (Micro-CT) with bone marrow density (BMD) calculations.

### Light microscope observation of the regenerated calvarial defect

To determine the histological features of the regenerated bone in the calvarial defect, every specimen was fixed in 10% buffered formalin, decalcified in 4% nitric acid, and embedded in paraffin. The cross-sectioned area of the frontal bone containing the perforated area was microsectioned to a thickness of 4 μm and subsequently stained by hematoxylin and eosin [5]. The cross-sectional microsections of the regenerated bone were observed under a light microscope and the histological images were captured by a DP-70® digital camera (Canon Co., Saitama, Japan).

### Statistical analysis

All of the data above were analyzed and compared by analysis of variance (ANOVA ) using SPSS for Windows® (Version 12.2, SPSS Inc., USA) and mean values with 95% confidence intervals. Correlations between three different kinds of bone, and between 12 different irradiated conditions were analyzed by the Pearson's correlation test (*p* < 0.05).

## Results

### Electron beam irradiation to a total of 1,152 specimens

A total of 16 bone materials, including four allogenic, six xenogenic, and six synthetic bones, were irradiated under 12 different conditions, such as with 1.0 or 2.0 MeV, and at 60 or 120 kGy. Each bone material was divided into six specimens for the *in vitro* study and graft procedures, so a total of 1,152 specimens were irradiated by the electron beam (Figure 2).

### *In vitro* analyses after EBI

Specimens were examined to determine that they had no specific characteristics such as discoloration, broken powder, or coagulation. The macroscopic structures before and after EBI did not differ.

**Figure 5:**
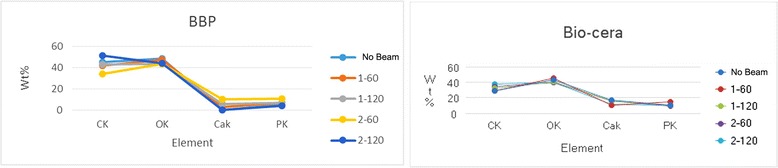
Elementary analysis of BBP® and Bio-cera® according to the different conditions of EBI.

**Figure 6:**
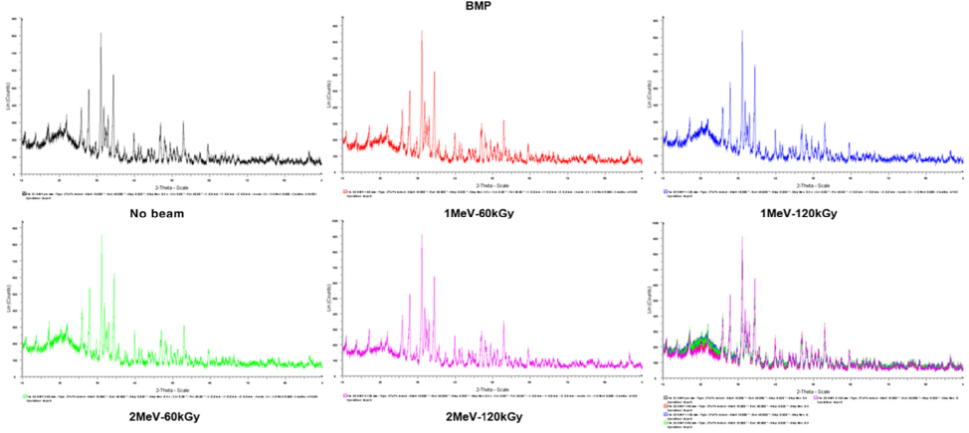
The X-ray diffraction (XRD) patterns of BMP® with several strong X-ray deflection peaks noted not to change with different EBI conditions.

In the elementary analysis of the allogenic and synthetic bone materials, no significant difference was found in the carbon, oxygen, calcium, and phosphorus components. In the BBP® and Bio-cera® allogenic bone materials, some differences according to the irradiaton conditions were found, but were not significant (Figure 5). The XRD patterns of the allogenic, xenogenic, and synthetic bone were identical, with several strong X-ray deflection peaks that did not differ with different conditions of electron beam energy or irradiation dose (Figure 6). By the three-dimensional construction of less-than-0.1-mm sized continuous optical sections, each specimen had their own CLSM images constructed. There were no differences observed before and after EBI in each specimen.

### Clinical evaluation and BMD calculations

We compared the specimens for possible clinical differences before and after EBI, and each bone material was implanted onto the perforated calvarial defect of the rats [6,7]. Clinically, there was no swelling, redness, or local infection around the suture site in all animals. No rats were lost in the interval period and all had normal-appearing weight, feeding, and activity during the 8 or 16 weeks.

**Figure 7:**
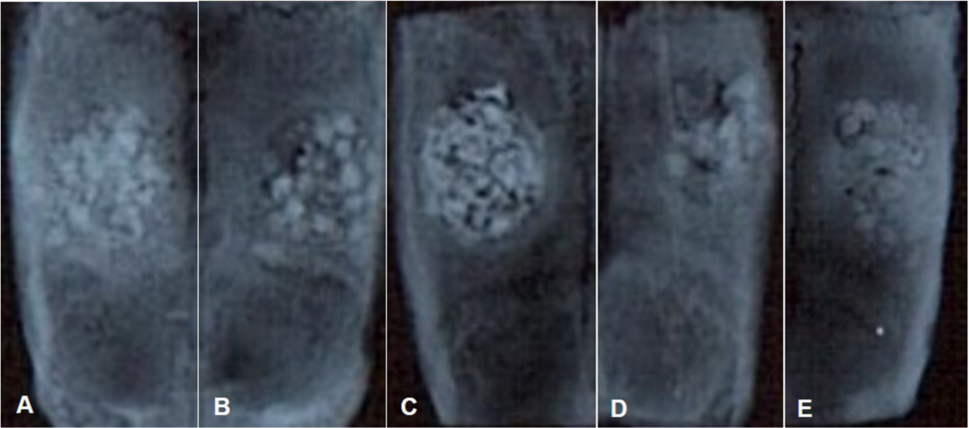
Horizontal radiographic views of the BMP® implanted frontal bone after 8 weeks, no beam treatment **(A)**, 1 MeV-60 kGy **(B)**, 1 MeV-120 kGy **(C)**, 2 MeV-60 kGy **(D)**, and 2 MeV-120 kGy **(E)**.

**Figure 8:**
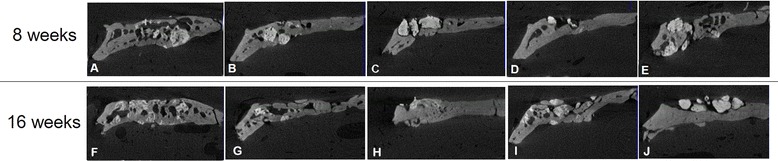
Comparison of the coronal Micro-CT views of the regenerated frontal bone under the different conditions after 8 and 16 weeks (right) of the electron beam-irradiated BMP® implantation. No beam treatment **(A,F)**, 1 MeV-60 kGy **(B,G)**, 1 MeV-120 kGy **(C,H)**, 2 MeV-60 kGy **(D,I)**, and 2 MeV-120 kGy **(E,J)**.

**Figure 9:**
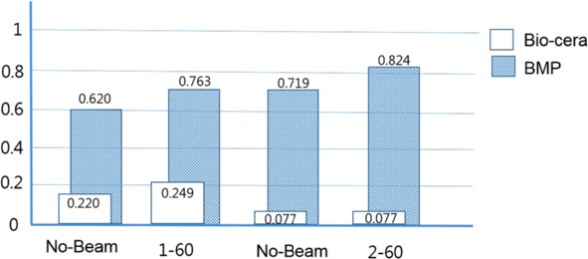
Comparison of bone marrow density in the Bio-cera® and BMP® bone materials, showing a slight increase in the 1 MeV-60 Gy and 2 MeV-60 Gy irradiated BMP®.

Incomplete bony healing was found in the horizontal radiographic view in most animals after 8 weeks under different electron beam energy and radiation conditions (Figure 7). There was less radiopacity observed in the 16 weeks specimen as compared to the 8 weeks specimen. No remarkable changes were found according to different electron beam energy or irradiation dose. In the comparison of the coronal Micro-CT views of the regenerated frontal bone under each irradiation condition after 8 and 16 weeks, many of the electron beam-irradiated xenogenic and synthetic bone materials become hard and dense after 16 weeks of observation (Figure 8). The BMD after 16 weeks was calculated to be higher than that after 8 weeks in most of the synthetic bone materials, but this pattern was not typical in the allogenic and xenogenic bone materials. This result suggested that any remaining bone particles did not undergo osteogenesis. For example, the BMD of BMP® was statistically higher under 1 MeV-60 kGy and 2 MeV-60 kGy conditions, but xenogenic bone such as Bio-cera® showed no special characteristics and did not show statistical significance (Figure 9).

### Light microscopic observation of the regenerated calvarial defect

**Figure 10:**
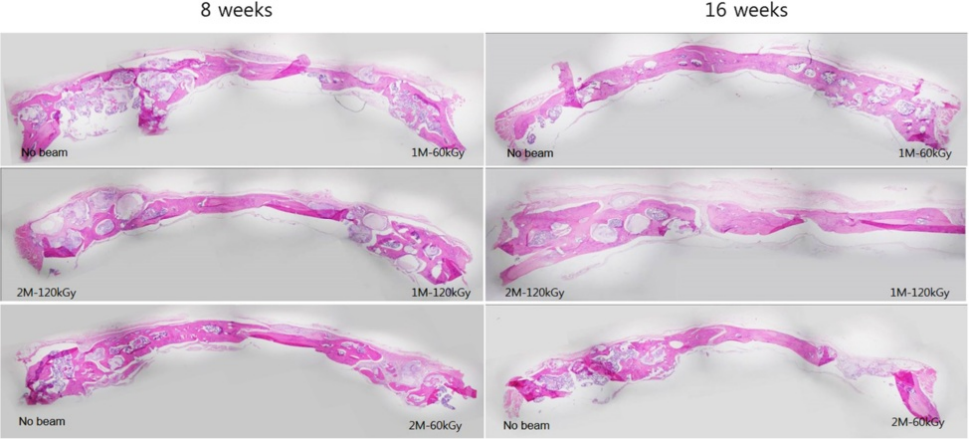
Photomicrographs of the regenerated frontal bone after 8 (left) and 16 weeks (right) after electron beam-irradiated BMP® implantation, with the coronal views of each condition are compared.

To determine the histological features of the regenerated bone in the calvarial defect, every specimen was observed under the light microscope. Histologically, new bone formation was more active in the synthetic bone materials than in the allogenic or xenogenic bone materials. The allogenic bone materials showed slightly more regenerative activity than the xenogenic bone materials. The BMP® grafted bone showed the most regenerative tendencies with bone marrow calcification at 8 weeks. However, there were neither remarkable differences, nor statistical significance in the samples between 8 and 16 weeks (Figure 10).

## Discussion

The application of nanotechnology in biomaterials engineering is one of the fastest growing areas in tissue engineering [8,9]. Radiation has been shown to be a useful tool to arrange atoms and ions with ion or electron beams. All radiation stems from ionizing radiation that can originate either from a radioactive source or from highly accelerated electrons [9-11].

For medical radiotherapeutical or engineering purposes, the most common form of radiation involves photons, with energies up to KeV and MeV and electrons with energies in the range between 4 and 15 MeV. In an X-ray tube, the cathode produces electrons by thermionic emission and acts as an electron source, with these electrons impinging on the positively charged anode after being accelerated in a strong electric field. The kinetic energy of the electrons is converted into X-radiation and then bremsstrahlung upon deceleration. The anode is composed of a high atomic-number material with a large bremsstrahlung cross-section and a high probability for producing bremsstrahlung, with about 99% of the kinetic energy of the electrons striking the anode transformed into thermal energy [8,9].

Charged particles produces electromagnetic radiation when they interact with matter, which is emitted as a characteristic line spectrum with energies typical for the emitting element, as well as bremsstrahlung with a continuous spectrum. Because of their large e/m (charge divided by mass) ratio, which is much greater than the e/m ratio for other charged particles like protons, deuterons or heavier ions, electrons produce significantly more bremsstrahlung. For commercial use, the most important characteristics for an accelerator are its electron energy and average beam power. Industrial electron accelerators are usually classified according to their energy range, which is classified as low (80-300 keV), medium (300 keV - 5 MeV), and high (above 5 MeV) [8,9].

**Figure 11:**
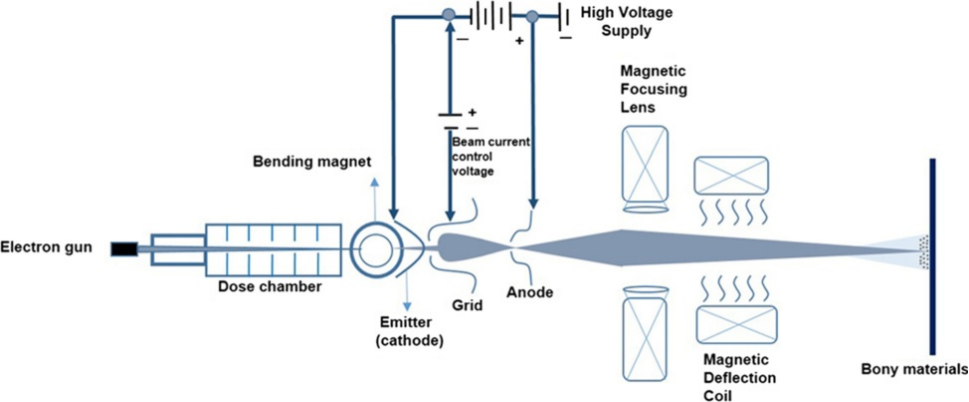
Schematic drawings of the basic electron beam accelerator including the electron gun, cathodic emitter, grid, anode, magnetic focusing lens and magnetic deflection coil. This accelerator includes the beam-defining system in electron mode.

EBI or electron beam processing is a process that involves using electrons, usually of high energy, to treat an object for a variety of purposes under elevated temperatures and a nitrogen atmosphere. EBI is also used to treat products with a high-energy electron beam accelerator which utilizes an on-off technology with a common design similar to that of a cathode ray television. Electron energies typically fall within the keV to MeV ranges, depending on the depth of penetration required. The basic components of a typical electron beam processing device are an electron gun, dose chamber, magnet, emitter, grid, anode, and deflection coil [1,3]. The electron gun is used to generate and accelerate the primary beam, while the magnetic optical focusing lens and deflection coil are used for controlling the way in which the electron beam impinges on the specimens (Figure 11). The cathode emitter is a source of thermally-emitted electrons that are both accelerated and shaped into a collimated beam by the electrostatic field geometry established by the grid and anode. The electron beam then emerges from the gun assembly through an exit hole in the ground-plane anode with an energy equal to the value of the negative high voltage being applied to the cathode. This use of a direct high voltage to produce a high-energy electron beam allows the conversion of input AC power to beam power at a greater than 95% efficiency, making electron beam material processing a highly energy-efficient technique. After exiting the gun, the beam passes through an electromagnetic focusing lens and magnetic deflection coil system. This focusing lens is used for producing either a focused or defocused beam spot on the specimens, while the deflection coil is used to either position the beam spot on a stationary location or provide some form of oscillatory motion [1,3,8].

EBI technology has been used in the engineering and manufacturing industry, mainly for product modifications. The first modification is the cross-linking of polymer-based products to improve their mechanical, thermal, chemical properties. The second purpose is for degradation, often used in the recycling of materials. The third purpose is for the sterilization of medical and pharmaceutical goods.

Cross-linking is the interconnection of adjacent long molecules with networks of bonds induced by chemical treatment or EBI. The cross-linking of polymers through EBI changes a thermoplastic material into a thermoset material making the polymer stable against heat by the impedance of molecular movement [5]. The benefits of cross-linking is due to the locking effect of molecules, which improves several properties including thermal resistance to high and low temperature, resistance to aging, mechanical strength (including tensile, modulus, abrasion resistance and creep resistance), chemical stress crack resistance, and heat shrink memory properties. Commonly cross-linked polymers include polyvinyl chloride (PVC), thermoplastic polyurethanes and elastomers (TPUs), polybutylene terephthalate (PBT), polyvinylidene fluoride (PVDF), polymethylpentene (PMP), polyethylenes, polyamides and nylons, and ethylene copolymers such as ethylene-vinyl acetate (EVA) and ethylene tetrafluoroethylene (ETFE). An electron beam induces the chain scissioning and cross-linking of these polymers. This chain scissioning effect makes the polymer chains shorter and result in changes in the crystallinity and microstructure of the materials. In the *in vivo* results of this study, some of the synthetic bone powder showed changes in its crystallinity, suggesting that the microstructure was able to accommodate some vasculature.

Chain-scissioning, also known as polymer degradation, is the breaking apart of molecular chains to produce molecular sub-units. The main effect of EBI is to break these molecular chains and reduce molecular weight. Without the use of any chemicals, EBI creates a chain-scission process that has been used to create fine micropowders from uses ranging from scrap and off-grade materials to inks and coatings for the automotive industry [12,13]. In addition, the high-energy electron irradiation lowers the energy barrier for ferroelectric-paraelectric phase transition and reduces polarization hysteresis losses in polyvinylidene fluoride-trifluoroethylene copolymers, with some of the energy barrier between calcium phosphate particles changed to reduce polarization hysteresis after EBI. Unfortunately, the mechanism underlying this degradation process has not been shown in *in vitro* analyses with SEM, XRD, or CLSM [14].

For microbiological sterilization, EBI has the ability to break the DNA chains of living organisms, such as bacteria, resulting in microbial death and rendering the space they inhabit sterile. EBI has already been used for the sterilization of medical products, the development of aseptic packaging materials for foods, as well as for disinfectants. EBI's sterilization effect can also be applied in biomaterials, especially bone grafts. For sterilization, EBI has significant advantages over other methods of sterilization currently in use. The process is quick, reliable, and compatible with most materials, and does not require any period of quarantine following processing [15]. For some materials and products that are sensitive to oxidative effects, the radiation tolerance levels for EBI may be slightly higher than for gamma exposure. This is due to the higher dose rates and shorter exposure times of EBI, which have been shown to reduce the degradative effects of oxygen [16].

As a means of disinfection for pests and other pathogens, EBI could replace antiquated and environmentally unfriendly methods such as fumigation and chemical dipping. A significant advantages of EBI is the reduction of bacterial contamination without compromising distinctive flavors and sensory properties. Fruits, vegetables, grains and other food items can also be processed by EBI to control the population of fruit flies and other insects that use these commodities as a host for propagation. Given these properties, EBI can be readily used to advance the use of biomaterials.

CLSM or laser scanning confocal microscopy (LSCM) are recent technique for obtaining high-resolution optical images with depth selectivity. While a conventional microscope can see as far into the specimen as the light can penetrate. While CLSM can only see images one depth level at a time. The key feature of CLSM is its ability to acquire in-focus images from selected depths, a process known as optical sectioning [17,18]. Images are acquired point-by-point and reconstructed in a computer, allowing for the three-dimensional reconstructions of topologically complex objects, such as synthetic bone apatite.

Since many materials can form crystals, including salts, metals, minerals, semiconductors, and various organic and biological molecules, XRD has been fundamental in the development of many scientific fields. X-ray crystallography is a tool used for identifying the atomic and molecular structure of a crystal, in which the crystalline atoms cause a beam of incident X-rays to diffract into many specific directions [19]. By measuring the angles and intensities of these diffracted beams, a crystallographer can produce a three-dimensional picture of the density of electrons within the crystal. From this electron density, the mean positions of the atoms in the crystal can be determined, as well as their chemical bonds, disorder and various other characteristics [20]. In this study, no changes in the peaks or other diffractive patterns in the bone substitutes before and after EBI were observed with CLSM or XRD. The effect of EBI on these bone particles could not be observed, so different approaches to induce bony regeneration are needed.

Various studies have reported the effects of EBI on various woods and semiconductive metals, but few data are available regarding the biologic and clinical applications of EBI on biomaterials.

Rat calvarial defects have been used to evaluate bone regeneration and survey different materials before clinical application in humans. The rat calvarial defect protocol involves preparation, surgery, and several analyses of bone regeneration, which has been described in our previous publications [1,4,6]. A defect if 8 mm is generally accepted to be the critical size in the rat calvarial defect model, but smaller defects have been investigated in models with two defects per animal, allowing fewer animals to be required for a given study. This study used a model with two 5-mm sized defects per calvarium, which has been used to examine the healing of subcritically sized defects. The potential for interactions between these two adjacent defects was prevented by allowing at least a 1.5-mm distance from the middle sagittal suture line. The primary goal of this design was to compare the regeneration of the bony materials, so the natural regenerative capacity of these two defects had to be excluded from the rat model [7].

EBI has four main effects to bony materials. First, it induces the cross-linking of biphasic calcium phosphate bony apatite, with a thermal effect on its resistance to temperature changes, a mechanical effect although with no specific changes to the surface morphology or apatite structure, and a chemical effect with a slight increase in the calcium and phosphorous contents in the synthetic bone, among other effects of irradiation such as changes in the DNA structure. Second, chain-scissioning causes the degradation and breaking of bony apatite chains, such as type I collagen, inducing blood supply formation and and coagulation near the grafted bony substitutes. Third, EBI has also been implicated in rheological changes, especially in BMP-coated apatites. Finally, EBI results in microbiological sterilization, as previously described.

The four desired properties of bone graft materials are osteogenesis, osteoinduction, osteoconduction, and osteointegration. Osteogenesis is new bone formation that occurs from osteoprogenitor cells that are present in the graft, survive the transplant, and proliferate and differentiate to osteoblasts. Marrow elements provide the fusion bed with osteoinductive proteins, potential osteogenic cells, and a local blood supply that make osteogenesis possible. Osteoconduction entails the stimulation and recruitment of nearby undifferentiated mesenchymal stem cells to the graft site. The stem cells are triggered to differentiate into chondrocytes and osteoblasts on the graft site. The method of recruitment and differentiation occurs through a cascade of events triggered by graft-derived factors known as BMP -2, -4, and -7, which are members of the transforming growth factor-ß superfamily. These BMPs are present in the matrix of the graft and are accessed after the mineral content of the bone graft has been removed. In addition to the BMPs, other vital factors involved in healing include platelet-derived growth factors, fibroblast growth factors, insulin-like growth factors, granulocyte colony-stimulating factors, mitogens, and interleukins. Angiogenic factors, such as vascular endothelial-derived growth factor, are also present. Osteoconduction is the ingrowth of vascular tissue and mesenchymal stem cells into the scaffold structure presented by the graft material. This is an ordered process that results in the formation of new Haversian systems in a predictable pattern along the host-graft interface, which subsequently infuses into the graft material. Osseointegration is described as the bonding of the host and the graft material. This phenomenon is vital to graft survival. For the graft to be functional, an inadequate amount of new bone must exist in the graft and unite with the host bone [21-23].

There are no organic or protein-related factors in the xenogenic and synthetic bone materials. Calcified or mineralized bone matrix has no osteoinductive properties and the BMPs are encased by the bone minerals, so the content of the allograft directly affects how additional proteins or factors can be used at the grafting site. The osteoinductive property is elevated with decreasing mineral content in the graft, with growth factors more available for the stimulation of mesenchymal cells. EBI has been suggested to have demineralization effects by creating new vacant spaces or micro-sized voids in the bony apatite. Within this space, a new vascular supply can be created for both osteoinduction and osteoconduction. Although any reduction in mineral content decrease mechanical strength of the graft, the scissoning effect of EBI can mitigate this decrease in mechanical strength.

As we have previously reported [1,2], type I collagen contributes to mineral deposition, vascular ingrowth, and growth factor binding, which provides a favorable environment for grafed bone regeneration. Type I collagen has the potential for immunogenicity and has diminished structural integrity. EBI can induce bone apatite to be incorporated and combined with type I collagen and BMPs.

Xenografts are derived from genetically different species. One of the most used xenograft is bovine bone, with Bio-oss® being the best-known example. These de-proteinized bovine bone minerals have been treated so that all of their organic materials have been removed. The application of these bones results in a crystal structure that practically matches human cancellous bone. In this study, the bone samples after 8 and 16 weeks of Bio-cera® showed some differences after EBI, with particle sizes of 0.25 to 1 mm, which are the dimensions that are thought to promote osteogenesis. 75% of its volume of Bio-oss® is known to be contained in a porous scaffold, so this structure greatly increases surface area and results in a material that is useful for osteoconduction. However, due to its large porous nature, the initial stability of Bio-oss® remains an issue. Treatment of Bio-oss® with EBI can increase angiogenesis and enhance further new bone growth.

## Conclusions

The use of EBI on synthetic bone has thermal, mechanical, and chemical effects on the cross-linking of biphasic calcium phosphate apatites. Another effect of chain-scissioning is the degradation or breaking of bony apatite chains, which can induce blood supply formation and coagulation near the grafted bony substitutes.

## Availability of supporting data

Recent advances in the understanding of EBI have resulted in new therapeutic strategies designed to improve the cross-linking of polymer-based products, the degradation of recycled materials, and the sterilization of medical and pharmaceutical goods. Additional effects of EBI include rheological changes, especially in the BMP-coated apatites, and the sterilization of microorganisms for pathogen control, anti-fungal fumigation, disinfection, and immunization.
